# Co-Inhibitory Molecules – Their Role in Health and Autoimmunity; Highlighted by Immune Related Adverse Events

**DOI:** 10.3389/fimmu.2022.883733

**Published:** 2022-06-16

**Authors:** Stinne R. Greisen, Maithri Aspari, Bent Deleuran

**Affiliations:** ^1^ Department of Biomedicine, Aarhus University, Aarhus, Denmark; ^2^ Department of Rheumatology, Aarhus University Hospital, Aarhus, Denmark

**Keywords:** PD-1, checkpoint inhibition therapy, autoimmunity, co-inhibitory receptors, immune related adverse events, rheumatic diseases

## Abstract

Immune checkpoint receptors are key players in regulating the immune response. They are responsible for both generating an immune response sufficient to kill invading pathogens, balancing the same response, and protecting against tissue destruction or the development of autoimmune events. The central role of the co-inhibitory receptors also referred to as inhibitory immune checkpoints, including PD-1 and CTLA-4 has become especially evident with the cancer treatments targeting these receptors. Blocking these pathways enhances the immune activity, resulting in both an increased chance of cancer clearance, at the same time induction of immune-related adverse events (irAE). Some of these irAE progress into actual autoimmune diseases with autoantibodies and symptoms, undistinguished from the naturally occurring diseases. This review will take advantage of the lessons learned from immune checkpoint blockade and relate this knowledge to our understanding of the same pathways in naturally occurring autoimmune diseases, mainly focusing on rheumatic diseases.

## Introduction: Balancing Health and Disease

The immune system is unique when it comes to its ability to protect our body and maintain homeostasis. It can both defend us against foreign pathogens and at the same time recognize and accept self-antigens. This equilibrium is also essential in avoiding cancer and controlling aging. Owing to the complexity of this system, we seem to be only scratching the surface in our understanding of the plethora of factors involved in this balanced regulation.

For T cells, the evolvement of a healthy immune system starts in the thymus where CD4 and CD8 T cells undergo positive and negative selection, to ensure an optimal reactivity to foreign antigens, and high tolerance towards self (1). Once out of the thymus, these cells will use an array of mechanisms when encountering a foreign antigen. The innate immune system is the first line of defense, subsequently resulting in the activation and recruitment of T cells and finally initiating an adaptive immune response. For antigen presenting cells to successfully activate a T cell, the T cell will need a second signal through an immune checkpoint, or co-receptor, which will be upregulated upon T cell receptor signaling or by cytokines (2, 3). The activation of co-receptors can either lead to increased activation of the T cell or inhibit the activation resulting in a dampened response or even anergy. The outcome from the balanced signaling between the multiple co-receptors thus determines the fate of an antigen-activated T cell. Often, the presentation of a high-affinity antigen that is significantly different from self, will result in an upregulation of the co-stimulatory receptors (CSR), like CD28 or 4-1BB, leading to full activation of the T cell (4). If the presented antigen resembles self or is of low affinity, upregulation of co-inhibitory receptors (CIR), including programmed death-1 (PD-1) and cytotoxic T-lymphocyte-associated protein 4 (CTLA-4) will be more pronounced (5, 6). These pathways will cause downregulation of the T cell activation, including diminished cytokine production, less proliferation, and reduced motility (7). Failure in balancing this second signal may cause chronic infections, cancers, or autoimmune diseases. The innate immune system can also initiate a chronic inflammatory condition, often referred to as autoinflammation. This topic is, however, beyond the scope of this review (8). In the following, we will focus on the CIR, their ligands, and their role in the development of autoimmune diseases. We consider this with the lessons learned from cancer treatment using immune checkpoint inhibitors (ICI), resulting in various inflammatory conditions, referred to as immune-related adverse events (irAE).

## Results: The First 10 Years of Immune Checkpoint Inhibitor Therapy

**Figure 1:**
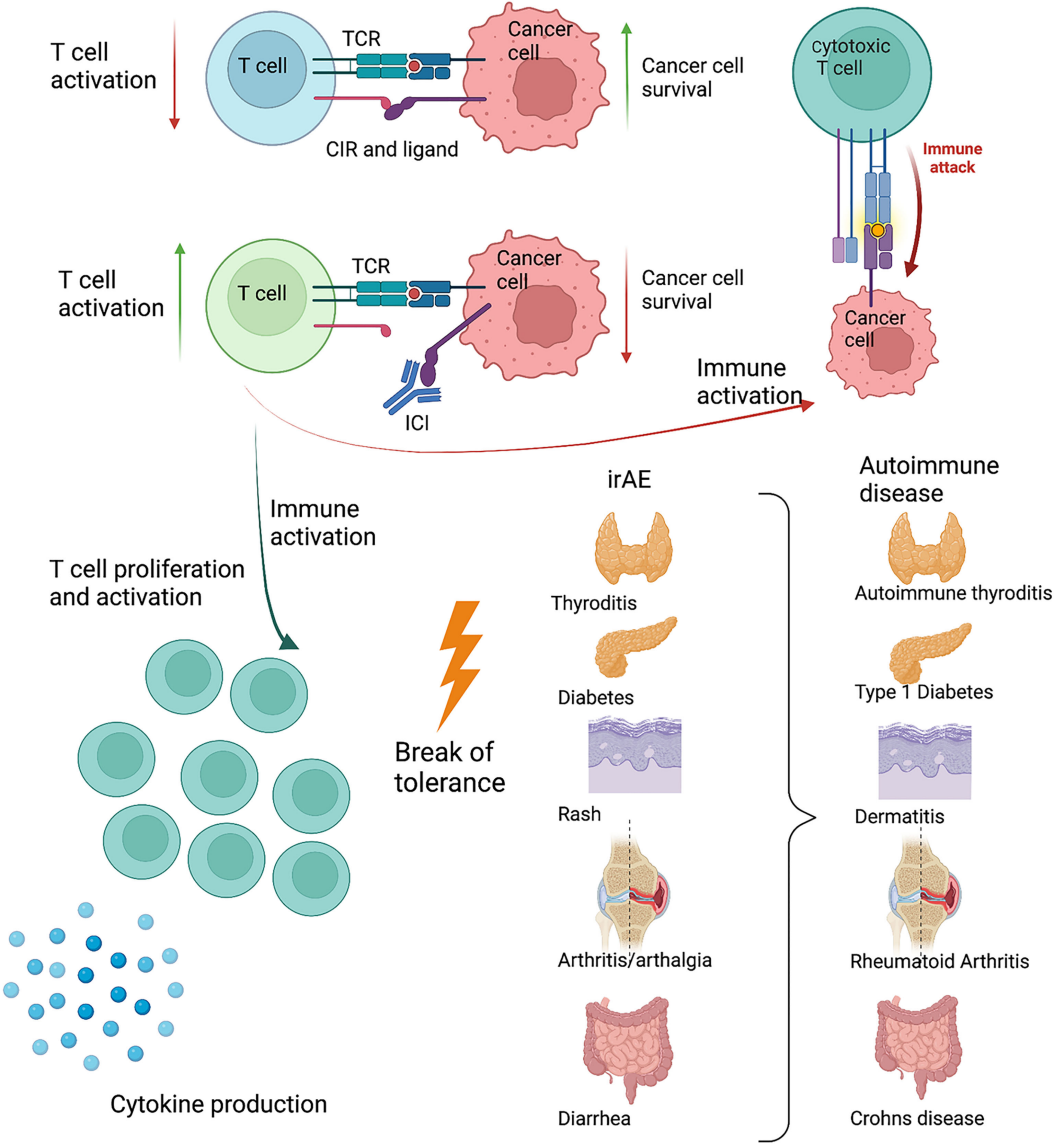
Schematic drawing of the interaction between an activated T cell and a cancer cell. The cancer cell exploits the PD-1 pathway, reducing T cell activity. When blocking the PD-1 pathway with antibodies, the T cell becomes sufficiently activated to kill the cancer cell. As a response to increased T cell activation and inflammation, immune related adverse events (irAE) develop. These target different organs and resembles known autoimmune diseases present in the same organ. Created with BioRender.com.

One of the central features in cancer development is the escape of immune surveillance. One mechanism exploited by cancer cells is the upregulation of ligands for the CIR, causing infiltrating immune cells to be shut down, thus avoiding immune mediated killing (9). Immune checkpoint inhibitors block CIR or their ligands. The first drug on the market targeted CTLA-4 (10, 11) and has in conjunction with ICI towards the PD-1 pathway, revolutionized cancer treatment. As a result of the increased immune activation, irAE and reduced cancer burden often goes hand-in-hand. However, these drugs provide valuable insight into the understanding of the role of CIR in the development of autoimmunity. In some cases, irAE become chronic conditions, closely resembling an autoimmune disease. Presentation with a full-blown debut of an autoimmune disease, even with autoantibodies is described (12, 13). IrAE are seen with a large variation in intensity, but are very common and affect 50%-70% of patients in monotherapy and more than 90% in combination therapy (12). Often, combination therapy targets both CTLA-4 and PD-1, which makes it difficult to pinpoint a specific clinical manifestation of one or the other. Despite several years in use, it remains difficult to predict which patients will respond to the treatment, and the search for prognostic biomarkers is ongoing (14). The development and treatment of irAE are often the governing steps for patients in ICI treatment. In patients with known systemic immunological disorders, ICI treatment is still limited to very few trials. Worsening, or flare in their disease seems to closely follow tumor regression (15). Considering the occurrence of irAE, it is appealing that the CIR pathways play a prominent role in the development of autoimmune diseases (**Figure 1**).

Many irAE tend to fade or disappear when the ICI treatment is ceased. These irAE thus deviate from what we normally see when examining an autoimmune disease. This supports a role for CIR serving to decrease immune activation, and their absence, or reduced function, leading to temporary symptoms or disease. Contrarily, upon considering the cases where the irAE progress into chronic diseases, these indicate that the dysfunction of CIR can induce a break in tolerance leading to autoimmunity (16). The factors determining these two different outcomes remain to be understood. More than 40% of the irAE develop into chronic conditions, often less responsive to steroids (17). The inflammatory conditions often present within the first 2-6 weeks of therapy, but may also arise already after the first treatment, or not until after several years of treatment (17, 18). Although autoimmune diseases are closely associated with HLA genotypes only a few studies have investigated the association between irAE and HLA type, and with no clear conclusion (19, 20).

It does occur that irAE are much more common than their “counter” autoimmune disease. One case is hypophysitis, an autoimmune reaction in the pituitary gland, very rare in its idiopathic form, but with an overall incidence after ICI treatment reported in a recent meta-analysis as 14%. This is a relatively common irAE, and also of significant severity, subsequently demanding life-long treatment as multiple hormonal axes are involved, and the damage is irreversible (21). Primary hypophysitis is not associated with systemic inflammation, and CIR have to our knowledge, not been investigated in the development of primary hypophysitis, where the mechanisms responsible for development are still largely unknown (22).

From a clinical viewpoint, it remains a challenge to treat patients with irAE especially if the symptoms become chronic, or hinder continued treatment of the oncologic disease. The first line of treatment is often corticosteroids, especially if these can be used locally (23, 24). However, systemic corticosteroid treatment results in both reduced antigen recognition and ability for cellular toxicity, especially ADCC. The use of systemic corticosteroid treatment thus raises a concern about the continued efficacy of the ICI to induce tumor eradication (25). This has resulted in a general consent to reducing corticosteroids to a minimum as quickly as possible, and a tendency to use disease-modifying anti-rheumatic drugs (DMARD) to reach control of the irAE. Anti-TNF antibodies and anti-IL-6R antibodies are the mainly used therapies, but large-scale and long-term studies are still not carried out (26, 27). Therefore, a better understanding of this area is needed, to improve treatment for both the cancer and the irAE.

## The CD28/B7 Family

**Figure 2:**
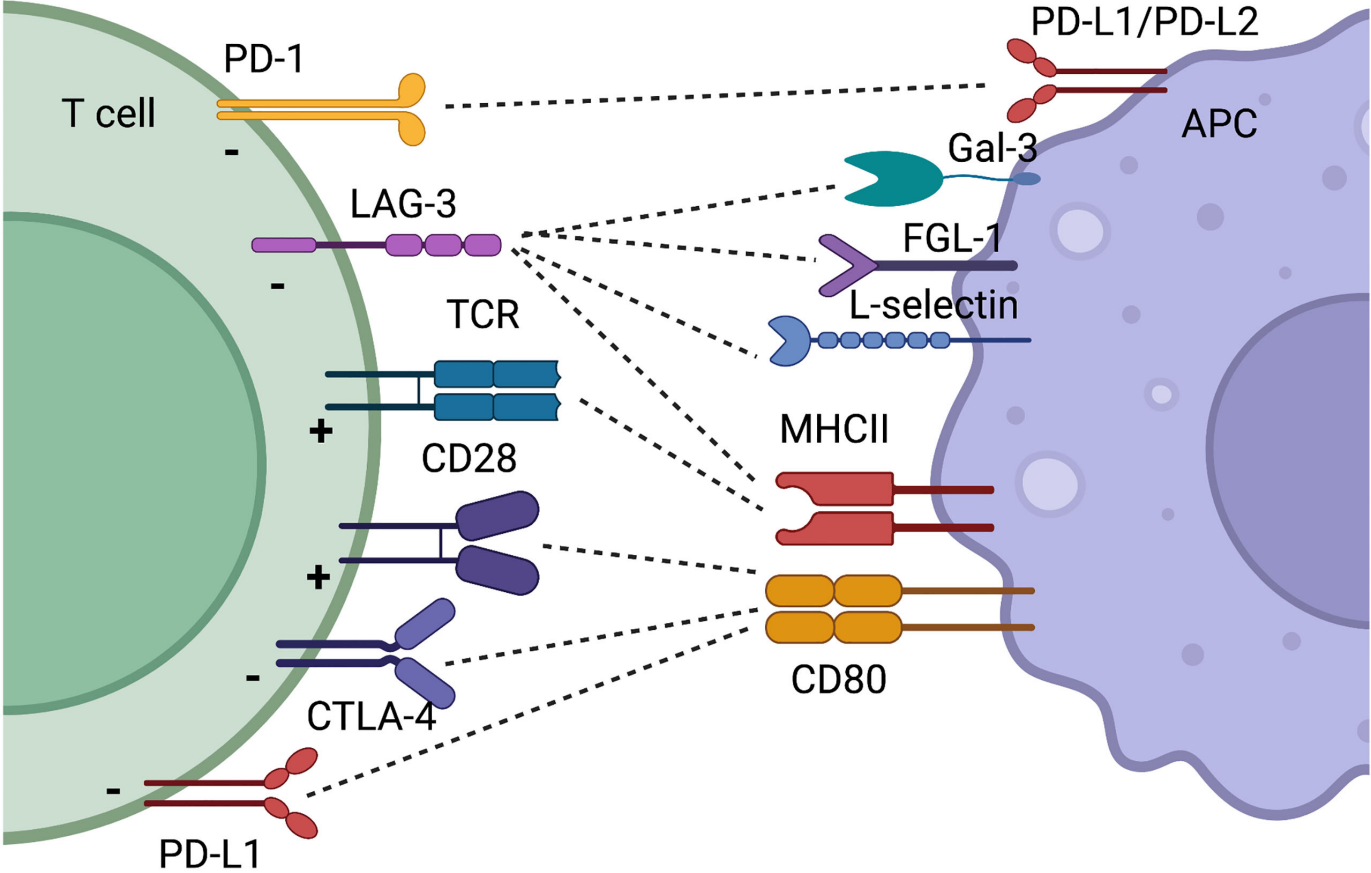
Simplified schematic illustration of CIR and CSR discussed in this review. Some receptors have binding partners which is illustrated by dotted lines. The function on the T cell by the receptor is indicated with a “+” for T cell activation and a “-” when T cell activity is decreased. Created with BioRender.com.

The CD28/B7 family of CSR and CIR is probably the best described among all co-receptors. CD28 was the first identified CSR and is crucial for optimal T cell activation (28). Signaling through the CD28 pathway induces complete T cell activation with increased motility, proliferation, and cytokine production including IL-2 and IFN-y. CD28 signals downstream through PI3K and PKC0, finally resulting in phosphorylation of AKT and NFkB. Mice lacking CD28 have a reduced immunoglobulin concentration and class switch, as well as decreased levels of IL-2. However, cytotoxic T cells do develop in these mice, and they do not succumb to infections (29). CD28 is necessary for the development of Tregs, but also favors the development of immune reactive T cells. Despite promising evidence (30), targeting CD28 in autoimmunity has not reached common clinical use, and especially for rheumatoid arthritis (RA), the focus has been on targeting CTLA-4, which also binds CD80, and competes with CD28 (31). This review will primarily focus on CIR, and the role of CD28 will not be discussed further. The two other two important members in the CD28/B7 family are CTLA-4 and PD-1, these being CIR (**Figure 2**).

## The PD-1 Pathway

PD-1 is a trans-membranous receptor mainly expressed by activated lymphocytes, however PD-1 expression has been described on several other cell types and in multiple tissues (32, 33). PD-1 has two ligands, PD-L1 and PD-L2 (34, 35). Engaging the PD-1 receptor causes phosphorylation of the ITSM motif and recruitment of SHP2, subsequently resulting in dephosphorylation of PI3K and AKT, eventually causing reduced activity in NFkB and thereof, a decreased T cell activation (3, 36, 37). Cancer cells utilize the expression of PD-L1 to silence inhibitory signals from cytotoxic T cells and other host immune cells. PD-1 or PD-L1 expression is often used as a marker (though poor) to identify tumors where treatment with ICI seems feasible (38). The PD-1 pathway not only signals through the PD-1 receptor; reverse signaling through PD-L1 is described, increasing cell survival and decreasing type 1 IFN responsiveness (39). This can be exploited by cancer cells, further supporting tumor growth and immune avoidance. PD-L1 reverse signaling is also crucial for chemokine-mediated dendritic cell migration from the skin to the lymph node (40). The role of reverse signaling in autoimmunity remains to be clarified.

Both PD-1 and its ligands are present in soluble (s) forms. These have been investigated as biomarkers of inflammatory diseases and multiple cancers. Most evidence suggests that levels of the soluble receptors increase as a response to inflammation. The functional role, if any, still remains up for debate. The soluble forms have been suggested to both increase immune activity by blocking the PD-1 pathway and decrease immune activity by positively engaging the PD-1 pathway. The role of the soluble forms is further complicated when recombinant proteins are used as surrogates for the soluble forms in both *in vivo* and *in vitro* studies (41–44).

The PD-1 pathway is central in maintaining immunological balance (33) and knockout mice of the PD-1 receptor causes lupus-like disease at a late stage in life (45).

PD-1 inhibitors are better tolerated than CTLA-4 inhibitors, but irAE affecting the joints, lungs and thyroid are more common (12). Hypothyroidism is the most common endocrinopathy seen in 6-10% (46)whereas newly onset T1DM is seen in about 1% of patients. In the development of childhood T1DM, polymorphisms in the PD-1 receptor are associated with an increased risk of T1DM. Patients with newly onset T1DM have decreased numbers of circulating CD4+PD-1+ T cells, and PD-1 fails to upregulate on peripheral T cells (47). The PD-1 axis is only scarcely investigated thyroid autoimmunity, but polymorphism in the PD-1 gene is associated with an increased incidence of autoimmune thyroiditis (48). Increased numbers of PD-1 infiltrating T cells are also seen in autoimmune thyroiditis (49). With hypothyroidism being one of the most prominent irAE after PD-1 blockade, this pathway might be more important than previously considered in the development of thyroid autoimmunity.

Many patients also experience rheumatological toxicities and close to 40% of the patients develop signs of inflammatory joint pain (16). The rheumatological toxicities can develop into diseases with autoantibodies, and both RA, scleroderma-like disease (50, 51), polymyositis and Sjögrens are seen.

Especially blocking the PD-1 pathway can cause pneumonitis which is a severe irAE, with high mortality. Especially, does pre-existing pulmonary fibrosis increase the risk of anti-PD-1-related pneumonitis (52, 53).

In accordance with the development of rheumatological irAE the PD-1 pathway plays a significant role in several rheumatological diseases including; RA, lupus (SLE), polymyalgia, polymyositis, and SSc (54). In RA; we have reported PD-1 expression to be increased both in the synovium and in the peripheral circulation (55–57). The increased expression correlates with disease activity, probably representing an immune activation. Looking further into the cellular and peripheral populations, PD-1 expression is associated with a pathological T cell phenotype involved in B cell activation and plasma cell maturation, resulting in increased antibody production and disease severity (58, 59). However, PD-1 expression also characterize regulatory T cells (Treg) and Tex which both are associated with less immune activation, and a better prognosis in autoimmune diseases (32, 57).

Looking into SLE the picture remains complicated. Genetic variances in the PD-1 gene are considered related to the development of the disease (60), and PD-1 expression is upregulated and associated with disease severity (61, 62). Recent bioinformatic and multi-omics approaches have made it evident that PD-1 is expressed on multiple different cell types, which are not solemnly associated with immune down regulation (58). The distinction between the different cell types expressing PD-1 is especially important in the understanding of how the PD-1 pathway is associated with disease activity and severity in SLE, where it is demonstrated that the increased PD-1 expression is especially seen in T helper cell populations with more pro-inflammatory properties (63). This has become evident that especially T peripheral helper cells (Tph) cells are upregulated in SLE -supporting the close association with autoantibodies in this disease (64). Turning to Treg, the expression of PD-1 is not reported different in SLE patients, however, the expression of PD-L1 is suggested decreased in SLE Treg, and in accordance is negatively correlated to disease activity (65). Despite lupus-like disease being one of the clinical manifestations in PD-1 KO mice, SLE as an irAE is relatively rare (66). It could be hypothesized that diseases largely driven by auto antibodies will take a long time to develop. When more patients will initiate early treatment with ICI, potentially increasing both survival and treatment duration, SLE-like disease could become a more common irAE.

Systemic scleroderma (SSc) is also a rheumatic disease, where autoantibodies play a significant role. Again, few cases have been reported after ICI treatment (12, 67). In SSc, a dysfunctional PD-1 pathway has been correlated with disease outcomes and clinical parameters (68). Raised levels of sPD-1 and sPD-L2 has been associated with increased skin thickness. In SSc patients, the numbers of PD-1–expressing cells within the Treg cell subset and within γδT cells (69) were significantly increased compared to those of healthy subjects. Increased frequency of T lymphocytes co-expressing PD-1 and T cell immunoreceptor with Ig and ITIM domains (TIGIT) was also observed in SSc patients. This accumulated expression of multiple CIR is in accordance with the hallmarks of exhausted T cells (Tex).

In *in vivo* models of mice with Topoisomerase I(Top-I)–induced SSc, production of IL-10 by Top-I specific B cells in cultures with T cells and Top-I protein was significantly higher than that by conventional B cells, this effect could be overcome by injection with recombinant chimeric PD-1-Fc and PD-L2-Fc (68), thereby supporting activation of the PD-1 pathway. Thus, substantiating the regulatory effect of PD-1 in maintaining homeostasis. Suggesting that interaction of PD-1 and PD-L2 is required for the production of IL-10 by B cells during T cell–B cell autoantigen-specific cognate interactions in SSc.

Taken together, it is clear that signaling through the PD-1 receptor decreases immune activation, and supports immunological tolerance. However, PD-1 is expressed both by exhausted T cells, but also as a result of activation. Therefore, PD-1 can be expressed by T cell driving immune activation, but also by T cells participating in lowering the immune reaction. This adds to the complexity of the understanding of when and how PD-1 expressing T cells are associated with disease activity in inflammatory conditions.

## CTLA-4

CTLA-4 is a transmembranous receptor expressed by activated T cells. Expression is upregulated upon T cell antigen encounter, and as for PD-1, low affinity antigens will induce a higher upregulation of CTLA-4. CTLA-4 binds to CD80/CD86 and competes with CD28 upon this binding, but with a higher avidity and affinity than CD28. A signal through CTLA-4 decreases T cell activation by the SHP2 and PP2A pathways, with many similarities to the PD-1 signaling pathway (70).

CTLA-4 is expressed by T cells, B cells, NK cells and regulatory T cells (Treg) (71, 72). T cells only express low levels of CTLA-4 on their surface, even when activated. CTLA-4 is mainly localized intracellular, where CTLA-4 is found in the golgi network, as well as in endosomes, secretory granules, and lysosomal vesicles from where it is circulated to the surface. This process is reviewed by Schneider et al (73).

CTLA-4 is also present in a soluble form, however, the importance of this is still not fully elucidated. Studies do support that sCTLA-4 plays an important role in keeping optimal immune surveillance (74).

In CTLA-4 knock-out mice, severe lymphoid proliferation and death appear, when the animals are 3-4 weeks old (75). Although no disease equivalent is known in humans, the importance of CTLA-4 in keeping tolerance is supported by fact that polymorphisms in the gene are associated with an increased risk of autoimmune disease. Importantly, these are not allocated to one disease but include T1DM, thyroiditis, Mb Addison, Crohn’s disease, RA, and multiple sclerosis, among many others (76). Engaging this pathway with CTLA-4:Ig it has been shown to be an effective treatment, highlighting that this pathway plays a role in maintaining immune reaction during autoimmune disease.

Antibodies targeting CTLA-4 are approved in multiple cancers including; metastatic melanoma, hepatocellular, renal carcinoma, mesothelioma, colon cancer, and non-small cell lung cancer, and more will be included in the near future. Monotherapy targeting CTLA-4 is associated with more severe irAE compared with monotherapy targeting the PD-1 pathway (12). This aligns with CTLA-4 KO mice having a much more severe phenotype than PD-1 KO mice (45, 75). The most common irAE from anti-CTLA-4 treatment are related to the gut and skin. This suggests CTLA-4 has a functional implication in preserving gut tolerance and supporting barrier functions (13, 77). Considering the evolving evidence of the major significance of the microbiota in the development of autoimmune diseases, including both rheumatic and gastrointestinal diseases, the role of CTLA-4, in combination with PD-1 is further highlighted by both their abilities to influence IgA synthesis and the microbiome (78, 79). Controversially, the CTLA-4:Ig fusion protein, Abatacept, was shown not to be clinically relevant in controlling Crohn’s Disease or ulcerative colitis in a clinical trial (80) . This does not exclude that CTLA-4 play an important role in keeping optimal conditions in the non-inflamed gut. This notion is supported by the observation that genetic changes in CTLA-4 is associated with an early onset of Crohn’s disease (81).

CTLA-4 functions in humans are elucidated by the *in vivo* use of CTLA-4-Ig fusion protein, used for the treatment of various immunological diseases. Abatacept was the first CTLA-4-Ig molecule to be approved for humans and has since been followed by Belatacept, with a higher affinity for CD86, making it 10-fold more potent *in vitro*, than Abatacept (82). These two CTLA-4-Ig molecules have been investigated in multiple autoimmune disorders, including RA, T1DM, diffuse cutaneous systemic sclerosis (dcSSc), psoriasis, and SLE. Both have also been tested, as inducers of tolerance to specific antigens upon organ transplantations. Especially Belatacept has shown to be efficacious in renal transplantation (83).

RA is by far the disease with the highest number of reports, as Abatacept has been approved for the treatment of moderate to severe disease for several of years (84). Its efficacy is in line with anti-TNF-antibodies (85). Since anti-CTLA-4 antibodies are used to treat cancer, it is tempting to suspect that treatment of RA with Abatacept would increase the risk of developing malignancy. However, no clear picture emerges. Some studies have reported a small increased risk, others none (86–88).

This naturally led to the question of why treatment with CTLA-4:Ig in RA, does not result in a greater increase in their cancer risk when blocking CTLA-4 in cancer results in autoimmunity. RA is dominated by a high number of both Tex cells and senescent T cells (89, 90), similar to chronic infections diseases. In patients with chronic hepatitis C, antiviral treatment lead to a decreased number of Tex cells (91). Assuming that Abatacept will do the same in RA the risk of developing cancer will also decrease as Tex favors cancer development. Therefore, it is a balanced outcome of disease activity, disease length, and treatment, thus explaining the low or absent risk of developing cancer in the Abatacept studies. The understanding of CTLA-4’s role in RA mainly comes from studying the effects of Abatacept. One major point of action shown for Abatacept is the downregulation of cytokine production in co-cultures, resulting in significantly lower concentration of IL-2, TNF-a and IL-1b (92). Additionally, data suggest that macrophages shift from an M1 to an M2 phenotype, when cultured in the presence of Abatacept (93), favoring a less inflammatory environment.

Fully in line with CTLA-4 function, Abatacept treatment slightly decreases but does not completely inhibit a vaccination response (94, 95).

In SLE and other inflammatory connective tissue diseases, Abatacept has shown efficacy in animal models, but generally failed in clinical studies (96, 97). Since the first casuistic report of Abatacepts efficacy in SSc, a phase II study has supported CTLA-4s possible role in the treatment of this disease (98, 99). Evidence on CTLA-4’s role in initiating connective tissue diseases remains to be fully elucidated, and most evidence is only on the genetic polymorphisms of CTLA-4 being associated with an increased risk of connective tissue diseases (100).

## LAG-3

Lymphocyte activation gene 3 (LAG-3) is still one of the less described CIR. LAG-3 is expressed upon T cell activation and has high homology with CD4. LAG-3 only binds to stabilized MHC class II molecules, suggesting that this inhibitory pathway exerts its functions when the T cells encounter antigens with a high specificity (101–103). Several other ligands for LAG-3 have been described. Among these, are Fibrinogen-like Protein 1 (FGL-1), L-selectin, and Galectin-3 (Gal-3) (104–106). As LAG-3 is also present in a soluble form, these multiple ligands and potential large multimeric formations suggest that the functionality of LAG-3 is complex. LAG-3 regulates T cell proliferation and homeostasis of both effector T cells and Tregs (107, 108).

Single LAG-3– and PD-1–deficient mice display minimal immunopathologic sequelae, double LAG-3/PD-1 knockout mice develop lethal systemic autoimmunity. Though mice lacking LAG-3 do not develop spontaneous autoimmune disease in non-autoimmune prone mouse strains, LAG-3 induced lethal myocarditis in BALB/c mice deficient for the gene encoding for PD-1. In addition, LAG-3 deficiency alone accelerated T1DM in nonobese diabetic(NOD) mice (10). Moreover, a cytotoxic LAG-3 Ab has been evaluated in a nonhuman primate model of delayed-type hypersensitivity (109). Contrarily, in a phase I clinical trial investigating psoriasis the depletion of LAG-3–positive T cells was linked to a reduced Th1-driven skin inflammation, lasting even in the absence of the depleting antibody (110).

The LAG-3 pathway is shown to be involved in the development of irAE such as colitis, RA, and diabetes (111, 112). Evidence from mouse studies suggests efficacy in cancer treatment, especially in combination with either anti-PD-1 or anti-CTLA-4 therapy. This has led to a recent study evaluating the effects of anti-LAG-3 antibodies in combination with anti-PD-1 antibodies, versus anti-PD-1 as monotherapy in patients with advanced melanoma (113). Here, addition of anti-LAG3 increased to the progression-free survival, but also increased the rate of irAE. With special relation to rheumatology, arthralgia was the most commonly increased irAE induced after targeting LAG-3. This supports LAG-3’s role in joint diseases. Currently, drugs that either augment LAG-3’s effects or deplete activated T cells that express LAG-3 are under development for chronic inflammatory diseases (114).

Despite emerging clinical trials with both agonistic and antagonistic antibodies targeting LAG-3, it is important to recognize that the knowledge of LAG-3 in relation to autoimmune disease is still rather limited. We have reported that LAG-3 could play a role in juvenile rheumatoid arthritis, supporting that LAG-3 could be important for immunoreactions and immune maturation during infancy (115).

Apart from the immune checkpoint molecules described here, many more are known, but few of these have reached the level of clinical studies. Among these are 4-1BB, TIGIT, TIM-3, B7-H3, B7-H4, ICOS to mention a few. The next decade will lead to new and interesting descriptions of effects and irAE that will help us to a better understanding of the immune system.

## Conclusion

Integrating our knowledge of irAE provides a better understanding of the role of CIR in both the initiation and progression of inflammatory diseases. It is suggestive that CIR may participate in the break of tolerance, but also in keeping the diseases in a chronic state. Which additional factors are needed for a break of tolerance when CIR are blocked, remains to be understood. It has also become evident that not all autoimmune diseases are represented equally in the irAE spectrum. Apart from myositis and myocarditis, inflammatory connective tissue diseases like SLE and SSc are still not as common as arthritis and polymyalgia. Among endocrinopathies, the thyroid gland is much more commonly affected than the pancreas. The reasons for this could be plenty. One is the time perspective as ICI treatment has only been around for less than a decade, and both diabetes and connective tissue diseases may develop over a longer period of time. For connective tissue diseases, especially considering that autoantibodies are present years before the development of clinical symptoms (116–118), which suggests a prolonged period to induce the disease. Also, the presence of these autoantibodies may be crucial for initiating the disease, and targeting two CIR may not be enough for autoantibodies to develop. Future observational studies with long term follow-up could change this picture.

With ICI drugs coming to the market targeting different CIR, we might see a more specific organ preference, depending on the targeted CIR and thereby reflecting the pathology of the “natural” autoimmune disease. A blurred picture is starting to emerge, where we see a tendency for CTLA-4 blockade to induce gut affection more often than blockade of the PD-1 pathway. By contrast, PD-1 and LAG-3 blockade more often result in pneumonitis and joint affection (119).

Our knowledge of CIR role is mainly associated with adults. Our major expositor to different antigens is highest during the first part of life, supporting that CIR play distinct functions dependent on age. We do, however, still know very little of their function in the early stages of life.

Taken together, it is clear that we have only scratched the surface, when it comes to understanding the complexity immune checkpoint molecules have on development of diseases related to immunosurveillance.

## Author Contributions

All authors contributed equally to searching and implementing relevant literature and editing the manuscript. SG prepared the initial draft and BD served as the senior and last author. All authors contributed to the article and approved the submitted version.

## Funding

Funding was received from Aarhus University, Lundbeck Foundation (R-287-2018-1094) and Danish Rheumatism Association (A6443).

## Conflict of Interest

The authors declare that the research was conducted in the absence of any commercial or financial relationships that could be construed as a potential conflict of interest.

## Publisher’s Note

All claims expressed in this article are solely those of the authors and do not necessarily represent those of their affiliated organizations, or those of the publisher, the editors and the reviewers. Any product that may be evaluated in this article, or claim that may be made by its manufacturer, is not guaranteed or endorsed by the publisher.
